# Neutrophil-to-lymphocyte ratio is associated with mortality in critically-ill cirrhotic patients

**DOI:** 10.1186/2197-425X-3-S1-A688

**Published:** 2015-10-01

**Authors:** N Moreau, P Forget, X Wittebole, PF Laterre, D Castanares-Zapatero

**Affiliations:** Intensive Care Unit, Université Catholique de Louvain, Cliniques Universitaires Saint Luc, Brussels, Belgium; Anesthesiology, Université Catholique de Louvain, Cliniques Universitaires Saint Luc, Brussels, Belgium

## Introduction

The neutrophil-to-lymphocyte ratio (NLR) is an easily accessible biomarker predictive of poor outcome in a variety of diseases. NLR is recognized to predict survival in uncomplicated cirrhosis, or in patients listed for transplantation.

Whether NLR is associated with prognosis in patients with an acute complication of cirrhosis has not been assessed. Still, there is growing evidence that cirrhotic patients presenting a worsening in the clinical condition develop an acute inflammatory response leading to organ dysfunction and mortality.

## Objectives

We aimed to assess whether NLR could be a predictor for mortality in patients experiencing an acute complication of cirrhosis.

## Methods

We reviewed data from ICU-patients admitted for cirrhosis-related complications. NLR values were calculated at admission and at day 1. Survival was estimated with Kaplan Meier method and analysed with the log-rank test. Survival model was performed using Cox proportional hazards. Multivariable forward stepwise method was used. Multivariate logistic regression and Receiver-operating characteristic (ROC) curves were generated.

## Results

The cohort included 108 patients (72% male, median age 58, average MELD score 21). Main causes of admission were gastro-intestinal bleeding (37%) and sepsis (36%). Mortality was 43% at day 28 and 45% at 3 months. The median NLR was 10.3 at admission and 8.3 at day 1. The median NLR was lower in survivors at admission (8.6 *vs* 13;p = 0.04) and day 1 (6.2 *vs* 12.2; p < 0.001). Septic patients displayed higher median NLR (admission: 7.7 *vs* 14.6;p = 0.002 and day 1: 6.5 *vs* 14.9;p = 0.004).

A decrease of NLR-values was observed at day 1 in the survivors (8.6 to 6.2; p = 0.008) whereas it remains elevated in non-survivors (13 *vs* 12.2;p = 0.49). In septic patients, NLR was not different between survivors and non survivors at any time.

There was a relationship between increasing quartile of NLR at day 1 and mortality (p = 0.01). Univariate analysis emphasized risk factors: NLR at day 1 (HR:1.02 _95_CI:1.001-1.03), SOFA score (HR:1.28 _95_CI:1.15-1.42), MELD score (HR: 1.05 _95_CI:1.01-1.08), mechanical ventilation (HR: 4.6 _95_CI:2.3-5.4) and hemofiltration (HR: 3 _95_CI:1.7-5.4). Following multivariate adjustments, NLR at day 1 (HR:1.02 _95_CI:1.01-1.03), SOFA score (HR:1.16 _95_CI:1.04-1.3) and mechanical ventilation (HR: 3.5 _95_CI:1.7-7.4) remained associated with mortality. NLR at day 1 was identified as risk factor in multivariate logistic regression (OR: 1.1 _95_CI:1.01-1.11) and area under curve was 72% (p < 0.001). The most discriminant value was 6.6 (sensibility 85%- specificity 54%). Survival was different according this value between both groups (p < 0.05).

## Conclusions

NLR is associated with mortality in cirrhotic patients with acute complication.

Its early ICU evolution is of interest for detecting severe outcome.
